# Factors associated with anti-retroviral therapy adherence among patients living with HIV during the COVID-19 pandemic: A cross-sectional study

**DOI:** 10.3389/fpsyt.2022.824062

**Published:** 2022-09-14

**Authors:** Ketut Suryana, Hamong Suharsono, Agung Wiwiek Indrayani, Luh Nyoman Arya Wisma Ariani, Wayan Wahyu Semara Putra, Ni Made Dwita Yaniswari

**Affiliations:** ^1^Department of Internal Medicine, Wangaya Hospital, Denpasar, Indonesia; ^2^Department of Biochemistry, Veterinary Faculty, Udayana University, Denpasar, Indonesia; ^3^Department of Pharmacology and Therapy, Udayana University, Denpasar, Indonesia; ^4^Department of Dermatology and Venereology, Faculty of Medicine, Udayana University- Sanglah Hospital, Denpasar, Indonesia; ^5^Department of Pulmonology, Wangaya Hospital, Denpasar, Indonesia

**Keywords:** adult PLWH, COVID-19 pandemic, anxiety disorder, ART adherence, anti-retroviral therapy

## Abstract

**Background:**

The coronavirus disease (COVID-19) pandemic causes fear and anxiety symptoms on some vulnerable populations such as patients living with human immunodeficiency virus (HIV) (PLWH). Physical distancing (during consultation in the clinic) and isolation restrictions will likely have a negative impact on/disruption to all care continuum services of HIV diseases although healthcare services and access to anti-retroviral therapy (ART) have continued to operate.

**Objective:**

To investigate the factors associated with ART adherence among PLWH during the COVID-19 pandemic.

**Methodology:**

A cross-sectional study was conducted on 324 PLWH who had been on ART for at least 6 months between June 2020 and January 2021. A semi-structured questionnaire was used to interview participants to collect data on sociodemographic characteristics and other factors.

**Results:**

Of 324 PLWH taking ART, 264 (81.48%) had high adherence (≥95%) and 60 (18.52%) had low adherence (< 95%). Factors independently associated with high ART adherence were employment status (adjusted odds ratio (AOR): 0.030, 95% confidence interval (CI): 0.010–0.088; *p* < 0.001), type of antiretroviral (ARV) (AOR: 3.101, 95% CI: 1.137–8.456; *p* = 0.027), family support (AOR: 0.157, 95% CI: 0.052–0.475; *p* = 0.001), the perception that the COVID-19 pandemic negatively impacts the ability to attend clinics (AOR: 7.339, 95% CI: 1.46–36.79; *p* = 0.015), and the perception that the COVID-19 pandemic negatively impacts the ability to take ART (AOR: 10.611, 95% CI: 2.98–37.72; *p* < 0.001).

**Conclusions:**

During the COVID-19 pandemic, factors associated with high ART adherence among PLWH attending the Hospital of Wangaya in Denpasar, Bali, Indonesia were employment status, ART type [non-fixed dose combination (FDC)], family support, and the perception that the COVID-19 pandemic negatively impacts the ability to attend clinics and to take ART.

## Introduction

Anti-retroviral therapy (ART) has been shown to be effective in suppressing viral replication, altering the natural course of the disease, and lowering morbidity and mortality (1–4). Human immunodeficiency virus (HIV) infection and acquired immunodeficiency syndrome (AIDS) continue to be major global public health issues (3, 5–7). It is now understood that HIV infection is a chronic inflammation that is potentially treatable. As a result, new conditions associated with the chronicity of the disease, such as ART adherence, have emerged (8). ART adherence is critical to long-term treatment success, therefore therapy has become a major source of concern. High ART adherence can reduce morbidity and mortality and improve the quality of life of patients living with HIV (PLWH).

Severe acute respiratory syndrome coronavirus 2 (SARS-CoV-2) infection can cause a wide variety of symptoms, ranging from asymptomatic carriage to moderate flu-like symptoms to severe pneumonia and respiratory failure requiring admission to an intensive care unit (ICU) (9). Since the beginning of the pandemic, several publications have been published, both on the link between HIV and an increased risk of acquiring symptomatic SARS-CoV-2 infection and on the poor prognosis of the new 2019 coronavirus disease (COVID-19) in PLWH (10). The COVID-19 pandemic has now become a health and psycho-socio-economic threat, and potentially cause fear, anxiety, and distress among PLWH because of how COVID-19 may impact their lives (9). In Indonesia, a total of 2,135,998 COVID-19 cases had been confirmed as of 28 June 2021 (10). Following the Eid al-Fitr celebrations in May, Indonesia experienced a spike in COVID-19 cases nationwide, following a downward trend in cases and deaths in March. A significant increase in lodging occupancy rates in high-risk provinces since the beginning of June is a source of great concern. As of 28 June 2021, 19,616,389 exams had been conducted. By the end of June, daily testing had risen from roughly 50,000 tests/day in March to close to 74,000 tests/day. As of June, the national test positivity rate was 23.2 per 100,000 (10).

Access to ART is a critical public health priority during the COVID-19 pandemic. Improving adherence necessitates collaboration with patients to identify and address individual barriers to COVID-19 and adherence (11–14).

This study investigated factors associated with ART adherence among PLWH during the COVID-19 pandemic.

## Methodology

### Study population and design

A cross-sectional study enrolled PLWH who visited Wangaya Hospital, in Denpasar, Bali between June 2020 and January 2021 and were included in the study on the basis of (1) receiving ART for at least 6 months; (2) being willing to be interviewed; and (3) giving written or verbal informed consent. There were 324 participants who took part in this study. All PLWH should receive a psychosocial approach who gave written informed consent during the visit every 30 days on a routine basis. Participants were questioned about their sociodemographic characteristics and the length of time they had been taking ART. Age, gender, education, the status of employment/occupation, address (travel burdens), antiretroviral (ARV) type, support, and the perception that the COVID-19 pandemic has a negative impact on patient's ability to visit clinics, and the perception that the COVID-19 pandemic has a negative impact on the ability to take ART were the factors associated with ART adherence among PLWH. The GAD-7 questionnaire was used to assess anxiety disorders (ADs), with the sensitivity and specificity of this tool using the optimal cutoff point being 89 and 82%, respectively. The severity of AD was classified into two groups: non-severe AD, mild to moderate anxiety (GAD-7 score ≤ 10), and severe AD (GAD-7 score >10) (9).

### Adherence measurement

In this study, self-report was used to measure adherence. This method was considered to be simple, practical, inexpensive, flexible, and of short duration. It was also confirmed by pill counts, which consisted of determining the number of pills remaining in patients' bottles. Patients returned the pill bottles, so that clinicians could physically count the remaining pills. Pill counts were determined retrospectively using a 30-day recall. The adherence index was calculated by the formula (15):


$$\begin{array}{l} \frac{\text{Total number of drugs taken (for 30 days)}}{\text{Total number of drugs prescribed (for 30 days)}}\times \text{100} \end{array}$$

Patients living with HIV with ≥95% adherence were considered to have high adherence, while those with <95% were considered to have low adherence (16–19).

### Statistical analysis

The data were analyzed with the Statistical Package for Social Sciences (SPSS), statistics for Windows version 26.0. To characterize the study population status, we report numbers and proportion for categorical variables and means with standard deviation (SD) for normally distributed continuous variables and median with interquartile range for non-normally distributed variables. Bivariate analysis was used to calculate crude odds ratios (OR) with 95% confidence intervals (CIs) to investigate the relationship between the independence variables, including sociodemographic and other characteristics and ART adherence. The Chi-squared test or Fisher's exact test was used for categorical variables, *t*-test for normally distributed continuous variables, the Mann–Whitney *U*-test for non-normally distributed continuous variables, and the Kruskal–Wallis test for ordinal variables, and a *p* < 0.05 was considered statistically significant.

### Ethical clearance

The approval to conduct this study was granted by local ethical committees. Ethical clearance no 09/RSUDW/Litbang/2020 was obtained. The study was carried out in accordance with the Declaration of Helsinki. Patients who visited Wangaya Hospital, in Denpasar, Bali, Indonesia provide us with data, which we then analyzed. All participants were asked to give informed consent.

## Results

In this study, the baseline characteristics among 324 participants are included and are described in detail in Table 1. Overall, 81.48% (264/324) of the participants were reported with high adherence, but 18.52% (60/324) of the participants were reported with low adherence (Table 2).

**Table 1:** Characteristic data of the participants (*n* = 324).

**Variables**	***N* (%) / Mean ±SD**
Age (year)^*^	
<34	153 (47.2%)
≥34	171 (52.8%)
**Sex**	
Male	197 (60.8%)
Female	127 (39.2%)
**Education**	
Uneducated	6 (1.9%)
Elementary school	45 (13.9%)
Junior high school	58 (17.9%)
High school	186 (57.4%)
University	29 (9.0%)
**Employment status**	
Employed	250 (77.2%)
Unemployed	74 (22.8%)
**Address**	
In Denpasar	242 (74.7%)
Out of Denpasar	82 (25.3%)
**Type of ARV**	
FDC	166 (51.2%)
Non-FDC	158 (48.8%)
**Support**	
No support	102 (31.5%)
Family	116 (35.8%)
NGO	106 (32.7%)
**Anxiety disorder (AD)**	
Severe anxiety disorder (GAD-7: >10)	124 (38.3%)
Non-severe anxiety disorder (GAD-7: ≤ 10)	200 (61.7%)
**Measure assessed at post-lockdown assessment**	
Perception that COVID-19 pandemic negatively impacts ability to come to clinic	139 (42.9%)
Perception that COVID-19 pandemic negatively impacts ability to take ART	75 (23.1%)

* , Age in median.

**Table 2:** Associated factors to adherence levels among PLWH (*n* = 324).

**Variables**	**High adherence** ***N* = 264 (81.48%)**	**Low adherence** ***N* = 60 (18.52%)**	***p*-value**
Age (year)^*^			
<34	118 (44.7%)	35 (58.3%)	*p* = 0.03
≥34	146 (55.3%)	25 (41.7%)	
**Sex**			
Male	165 (62.5%)	32 (53.3%)	*p* = 0.12
Female	99 (37.5%)	28 (46.7%)	
**Education**			
Uneducated	4 (1.5%)	2 (3.3%)	*p* = 0.60
Elementary school	35 (13.3%)	10 (16.7%)	
Junior high school	45 (17.0%)	13 (21.7%)	
High school	155 (58.7%)	31 (51.7%)	
University	25 (9.5%)	4 (6.7%)	
**Employment status**			
Employed	234 (88.6%)	16 (26.7%)	*p* < 0.01
Unemployed	30 (11.4%)	44 (73.3%)	
**Address**			
In Denpasar	195 (73.9%)	47 (78.3%)	*p* = 0.29
Out of Denpasar	69 (26.1%)	13 (21.7%)	
**Type of ARV**			
FDC	120 (45.5%)	46 (76.7%)	*p* < 0.01
Non-FDC	144 (54.5%)	14 (23.3%)	
**Support**			
No supported	88 (33.3%)	14 (23.3%)	*p* < 0.01
Family	112 (42.4%)	4 (6.7%)	
NGO	64 (24.2%)	42 (70.0%)	
**Anxiety disorder (AD)**			
Severe anxiety disorder (GAD-7: >10)	79 (29.9%)	45 (75.0%)	*p* < 0.01
Non-severe anxiety disorder (GAD-7: ≤ 10)	185 (70.1%)	15 (25.0%)	
**Measure assessed at post-lockdown assessment**			
Perception that COVID-19 pandemic negatively impacts ability to come to clinic	90 (34.1%)	49 (81.7%)	*p* < 0.01
Perception that COVID-19 pandemic negatively impacts ability to take ART	174 (65.9%)	11 (18.3%)	*p* < 0.01

ARV, anti-retroviral; FDC, fixed dose combination; NGO, non-government organization; GAD, generalized anxiety disorders; COVID, coronary virus disease.

* , Age in median.

There is a statistically significant relationship between high adherence with older age (≥34-year old) (*p* = 0.03), employment status/employed (*p* < 0.01), ARV type (due to adverse effect) (*p* < 0.01), family support (*p* < 0.01), and non-severe anxiety (*p* < 0.01). The perception that the COVID-19 pandemic negatively impacts the ability to attend clinics and to take ART showed a statistically significant association (*p* < 0.01) (Table 2).

In this study, we discovered a statistically significant relationship between high ART adherence and older age, employment status/employed, ARV type (due to adverse effects), family support, education, no-severe anxiety, and the perception that the COVID-19 pandemic had a negative impact on the ability to visit clinics and to take ART but no association with sex, education, and address.

In the bivariate analysis, there was a statistically significant association between older age (≥34-year old) and high ART adherence (*p* = 0.03). Employment status/employed had a statistically significant association with high ART adherence (*p* < 0.01). We reported that the ARV type (due to the side effects of ARV) had a statistically significant relationship with high ART adherence (*p* < 0.01). Due to the perception that the COVID-19 pandemic had a negative impact on the ability to visit clinics and take ART, this study found a statistically significant association between the COVID-19 pandemic and high ART adherence (*p* < 0.01) (Table 2).

The multivariate analysis was done by logistic regression, and we found a statistically significant association between employment status (adjusted odds ratio (AOR): 0.030, 95% CI: 0.010–0.088; *p* < 0.001), the type of ARV (AOR: 3.101, 95% CI: 1.137–8.456; *p* = 0.027), support (AOR: 0.157, 95% CI: 0.052–0.475; *p* = 0.001), the perception that the COVID-19 pandemic negatively impacts the ability to come to clinics (AOR: 7.339, 95% CI: 1.46–36.79; *p* = 0.015), and the perception that the COVID-19 pandemic negatively impacts the ability to take ART (AOR: 10.611, 95% CI: 2.98–37.72; *p* < 0.001), with adherence levels among PLWH (Table 3).

**Table 3:** Multivariate analysis of adherence levels among PLWH (*n* = 324).

**Variables**	**AOR**	**95%CI**	***P*-value**
Age (years)	2.108	0.703–6.319	0.183
Anxiety disorders	0.732	0.154–3.493	0.696
Employment status	0.030	0.010–0.088	<0.01
Type of ARV	3.101	1.137-8.456	0.027
Support	0.157	0.052–0.475	0.001
Perception that COVID-19 pandemic negatively impacts ability to come to clinic	7.339	1.46–36.79	0.015
Perception that COVID-19 pandemic negatively impacts ability to take ART	10.611	2.98–37.72	<0.01

ARV, antiretroviral; ART, antiretroviral therapy, COVID-19, coronavirus disease 2019.

## Discussion

In the management of chronic diseases, including HIV infection, ART adherence is also a major issue (16, 20). According to HIV studies, high ART adherence is required for viral suppression, prevention of ART resistance and disease progression, and decreased risk of transmission (17–20). A variety of factors influence ART adherence, such as drug toxicity and regimen complexity (21, 22).

In this study, 324 participants were involved, of whom 81.48% (264 participants) reported high adherence (≥95% of their pills were taken in the previous 30 days) and 18.52% (60 participants) reported low adherence (<95% of their pills were taken in the previous 30 days). This was different from our previous study (prior COVID-19 pandemic); among 202 participants. Of these, 84.16% (170/202) had high ART adherence and 15.84% (32/202) had low ART adherence (23).

In this study, older age (≥34-year old) was not associated with high ART adherence (AOR = 2.108; 95% CI: 0.703–6.319, *p* = 0.183). On the other hand, Tolossa et al. (24) reported that the odds of non-adherence to ART were 3.41 times higher among patients younger than 25 years as compared to their counterpart (OR = 3.41, 95% CI: 1.26–9.21) (24).

Employment status/employed has a statistically significant association with high ART adherence (AOR = 0.030; 95%CI: 0.010–0.088, *p* < 0.01). Prah et al. (20) found that employment status/employed was significantly related to ART adherence (*p* < 0.01) (20). Also Safira et al. (16) reported that employed had a statistically significant association with high ART adherence (*p* = 0.03) (16). Suleiman et al. (17) found that employment was significantly associated with high ART adherence (*p* < 0.01) (17). According to Ibrahim et al. (18), those who were unemployed were more likely to fail to comply with ART (*p* = 0.01) (18). According to Talam et al. (19), high ART adherence was associated with employment (*p* = 0.01) (19).

Furthermore, other factors contribute to the effectiveness of ART, such as participants' ability to follow particular instructions regarding dose intervals as a result of regular ARV use (25–27).

We reported that ARV type (due to the side effects of ARV) had a statistically significant relationship with high ART adherence (AOR = 3.101; 95% CI: 1.137–8.456, *p* = 0.027). Cheng et al. (27) found that ARV type (treatment side effect) was associated with high ART adherence (*p* < 0.01) (27). According to Achappa et al. (28), ART side effects were significantly associated with ART adherence (*p* < 0.01) (28). According to Oliveira et al. (1), adverse drug effects were associated with ART adherence (*p* < 0.01) (1). Shigdel et al. (29) found that the adverse effects of ARV were related to therapy adherence (*p* < 0.05) (29).

The support of family increases the patient's self-esteem, making it easier for the patient to stick to ART and suggest that the patient will be alive for a long time and in good health. In contrast, if a patient lacks support, the patient's situation becomes more difficult because what follows is ill-treated from the family, and the person loses hope, which leads to treatment failure (11). In this study, we found that family support was statistically significantly associated with high ART adherence (AOR = 0.157; 95% CI: 0.052–0.475, *p* = 0.001). Another study conducted by Achappa et al. (28) reported that good family support was statistically significantly associated with high ART adherence (*p* < 0.01). According to a meta-analysis, participants from cohesive families have 1.74 times higher adherence (28). Yathiraj et al. (30) discovered that participants with a strong family support (74%; OR = 3.0, CI = 1.2, 5.2) were more likely to adhere to ART (*p* < 0.05) (30). According to Joseph et al. (31), family support was statistically associated with high ART adherence (*p* < 0.01) (31).

Anxiety is an adaptive emotional and behavioral response to threatening stimuli that is necessary for survival, characterized by chronic and persistent worry. Factors that may be associated with anxiety include health, finances (due to job loss), physical distancing, social isolation, and school closures, as occurred during the COVID-19 pandemic (14, 32, 33).

This study reported no statistically significant association between high ART adherence and non-severe anxiety (AOR = 0.732; 95% CI: 0.154–3.493, *p* = 0.696). The study conducted in India reported that AD was found in 39.5% of 152 participants. Another study found that the lower proportions of AD 13.0% in Wuhan and 10.8% in Singapore (34–36). There was a significantly higher frequency of participants with anxiety vs. without anxiety in Bangladeshi during the COVID-19 pandemic (96.1 vs. 69.8%, *p* < 0.01) (33).

Due to the perception that the COVID-19 pandemic has negatively affected the ability to visit clinics and the ability to take ART, this study found a statistically significant association with high ART adherence (AOR = 7.339; 95%CI: 1.46–36.79, *p* = 0.015; AOR = 10.611; 95% CI: 2.98–37.72, *p* < 0.01, respectively). It is clear from this study that COVID-19 has adversely affected the capacity of many PLWH to attend the clinic and their ability to take ART. Client travel to HIV clinics during COVID-19 has become difficult due to the lack of transportation, staff checks during the lockdown, and the lack of funds to pay for transportation, and PLWH were afraid of being infected when visiting the clinic. Dorward et al. (37) reported that ART initiation reduced from a weekly median of 571 prior to lockdown (interquartile ranges (IQR) 498–678) to 375/week after lockdown (331–399), in the 1st week of lockdown, the Poisson regression model estimated to have decreased by 46.2% (30 March 2020 to 5 April 2020; IRR 0.538, 0.459–0.630) (37). Dear et al. (38) reported that during the early COVID-19 period, PLWH were less likely to be adherent to HIV clinic visits (adjusted OR: 0.64; 95% CI: 0.45–0.91); however, this association was not significant in the late COVID-19 period (AOR: 0.82; 95%CI: 0.57–1.19) (38).

In this study, we discovered no relationship between high ART adherence and gender, education, or address (travel burdens). Other studies reported no association between ART adherence and sex, education, or income (38). According to Byabene et al. (39), high ART adherence was not associated with time to the clinic (travel burdens) (*p* = 0.07) (39).

## Conclusion

Generally, participants have high ART adherence. There is a higher proportion/percentage of participants with high ART adherence in our previous study (before the COVID-19 pandemic) than during the COVID-19 pandemic. High ART adherence is statistically significantly associated with employment, the type of ARV being used, family support, and the perception that the COVID-19 pandemic negatively impacts the ability to attend clinics and to take ART.

## Limitations

This study had some drawbacks, including a small sample size. Between June 2020 and January 2021, this study was conducted in a single center at Wangaya Hospital in Denpasar, Bali-Indonesia. The use of a cross-sectional design limited the degree to which causal inferences and generalizations could be made from the research findings.

## Data availability statement

The raw data supporting the conclusions of this article will be made available by the authors, without undue reservation.

## Ethics statement

The studies involving human participants were reviewed and approved by Wangaya Hospital. The patients/participants provided their written informed consent to participate in this study. Written informed consent was obtained from the individual(s) for the publication of any potentially identifiable images or data included in this article.

## Author contributions

KS contributed to the concept of the research idea, study design, data analysis, and paper drafting. HS contributed to the design study and the revision of this paper. AI and LW contributed to the data collection, paper drafting, and the revision of this paper. WP and NY contributed to the data collection and the revision of this paper. All authors contributed to the article and approved the submitted version.

## Conflict of interest

The authors declare that the research was conducted in the absence of any commercial or financial relationships that could be construed as a potential conflict of interest.

## Publisher's note

All claims expressed in this article are solely those of the authors and do not necessarily represent those of their affiliated organizations, or those of the publisher, the editors and the reviewers. Any product that may be evaluated in this article, or claim that may be made by its manufacturer, is not guaranteed or endorsed by the publisher.

## References

[1] de Oliveira Costa J Schaffer AL Medland NA Litchfield M Narayan SW Guy R. Adherence to antiretroviral regimens in Australia: a nationwide cohort study. AIDS Patient Care STDS. (2020) 34:81–91. 10.1089/apc.2019.0278 32049558

[2] Silva MC Ximenes RA Miranda Filho DB Arraes LW Mendes M Melo AC. Risk-factors for non-adherence to antiretroviral therapy. Rev Inst Med Trop São Paulo. (2009) 51:135–9. 10.1590/S0036-46652009000300003 19551287

[3] Hornschuh S Dietrich JJ Tshabalala C Laher F. Antiretroviral treatment adherence: knowledge and experiences among adolescents and young adults in Soweto, South Africa. AIDS Res Treat. (2017) 2017:5192516. 10.1155/2017/5192516 28409026 PMC5376918

[4] Azia IN Mukumbang FC Van Wyk B. Barriers to adherence to antiretroviral treatment in a regional hospital in Vredenburg, Western Cape, South Africa. S Afr J HIV Med. (2016) 17:a476. 10.4102/sajhivmed.v17i1.476 29568618 PMC5843173

[5] Fonsah JY Njamnshi AK Kouanfack C Qiu F Njamnshi DM Tagny CT. Adherence to Antiretroviral Therapy (ART) in Yaoundé-Cameroon: association with opportunistic infections, depression, ART regimen and side effects. PLoS ONE. (2017) 12:e0170893. 10.1371/journal.pone.0170893 28141867 PMC5283684

[6] Miyada S Garbin AJ Gatto RC Garbin CA. Treatment adherence in patients living with HIV/AIDS assisted at a specialized facility in Brazil. Rev Soc Bras Med Trop. (2017) 50:607–12. 10.1590/0037-8682-0266-2017 29160506

[7] Kahema SE Mgabo MR Emidi B Sigalla GN Kajeguka DC. Factors influencing adherence to antiretroviral therapy among HIV infected patients in Nyamagana-Mwanza, Northern Tanzania: a cross sectional study. Int Arch Med Microbiol. (2018) 1:002. 10.23937/iamm-2017/1710002

[8] Hirsch JD Gonzales M Rosenquist A Miller TA Gilmer TP Best BM. Antiretroviral therapy adherence, medication use, and health care costs during 3 years of a community pharmacy medication therapy management program for Medi-Cal beneficiaries with HIV/AIDS. J Manag Care Pharm. (2011) 17:213–23. 10.18553/jmcp.2011.17.3.213 21434698 PMC10437600

[9] Choi E Hui B Wan E. Depression and anxiety in Hong Kong during COVID-19. Int J Environ Res Public Health. (2020) 17:3740. 10.3390/ijerph17103740 32466251 PMC7277420

[10] UNICEF. Indonesia COVID-19 Situation Report, June 2021(2021). Available online at: www.unicef.org. (accessed April 14, 2022).

[11] Adefolalu A Nkosi Z Olorunju S Masemola P. Self-efficacy, medication beliefs and adherence to antiretroviral therapy by patients attending a health facility in Pretoria, South African Family Practice. (2014) 56: 281–5. 10.1080/20786190.2014.975476

[12] Rajbhandari RM Karmacharya DB. Dixit SM. Strengthening adherence to Anti Retroviral Therapy (ART) monitoring and support: operation research to identify barriers and facilitators in Nepal. BMC Health Serv Res. (2015) 15:188. 10.1186/s12913-015-0846-8 25939593 PMC4428010

[13] Zegeye S Sendo EG. Adherence to antiretroviral therapy among HIV-infected children attending Hiwot Fana and Dil-Chora art clinic at Referral Hospitals in Eastern Ethiopia. J HIV Clin Scientific Res. (2015) 2:008–14. 10.17352/2455-3786.000007

[14] Lencha B Hasen K Getachew T Abdi M Habtamu M. Adherence to antiretroviral therapy and associated factors among people living with HIV/AIDS at Gobba hospital, Southeast Ethiophia: an institutional based study. Qua Prim Care. (2015) 23:336–41.

[15] Grymonpre RE Didur CD Montgomery PR Sitar DS. Pill count, self-report, and pharmacy claims data to measure medication adherence in the elderly. Ann Pharmacother. (1998) 32:749–54. 10.1345/aph.17423 9681089

[16] Safira N Lubis R Fahdhy M. Factors affecting adherence to antiretroviral therapy. KnE Life Sci. (2018) 4:60–70. 10.18502/kls.v4i4.2264

[17] Suleiman IA Momo A. Adherence to antiretroviral therapy and its determinants among persons living with HIV/AIDS in Bayelsa state, Nigeria. Pharm Prac. (2016) 14:631. 10.18549/PharmPract.2016.01.631 27011771 PMC4800010

[18] Ibrahim Y Sutan R Latif KBA Al-Abed AA Amara A Adam I. Poor adherence to antiretroviral therapy and associated factors among people living with HIV in Omdurman City, Sudan. Malaysian J Public Health Med. (2014) 14:90–101.

[19] Talam NC Gatongi P Rotich J Kimaiyo S. Factors affecting antiretroviral drug adherence among HIV/AIDS adult patients attending HIV/AIDS clinic at MOI teaching and Referral Hospital, Eldoret, Kenya. East Afr J Public Health. (2008) 5:74–8. 19024414

[20] Prah J Hayfron-Benjamin A Abdulai M Lasim O Nartey Y Obiri-Yeboah D. Factors affecting adherence to antiretroviral therapy among HIV/AIDS patients in Cape Coast Metropolis, Ghana. J HIV AIDS. (2018) 4:1–7. 10.16966/2380-5536.149

[21] Aragonés C Sánchez L Campos JR Pérez J. Antiretroviral therapy adherence in persons with HIV/AIDS in Cuba. MEDICC Rev. (2011) 13:17–23. 10.37757/MR2011V13.N2.6 21654587

[22] Adefolalu AO Nkosi ZZ. The complex nature of adherence in the management of HIV/AIDS as a chronic medical condition. Diseases. (2013) 1:18–35. 10.3390/diseases1010018

[23] Suryana K Suharsono H Antara IG. Factors associated with adherence to anti-retroviral therapy among people living with HIV/AIDS at Wangaya Hospital in Denpasar, Bali, Indonesia: a cross-sectional study. HIV/AIDS. (2019) 11:307–12. 10.2147/HIV.S219695 31819661 PMC6875560

[24] Tolossa T Wakuma B Mulisa D Besho M Tsegaye R Tigistu M. Adherence among people living with HIV seeking services from public health facilities in Western Ethiopia. HIV/AIDS. (2021) 13:1149–58. 10.2147/HIV.S336647 35002331 PMC8721927

[25] Letta S Demissie A Oljira L Dessie Y. Factors associated with adherence to Antiretroviral Therapy (ART) among adult people living with HIV and attending their clinical care, Eastern Ethiopia. BMC Int Health Hum Rights. (2015) 15:33. 10.1186/s12914-015-0071-x 26711659 PMC4693416

[26] O'Connor JL Gardner EM Mannheimer SB Lifson AR Esser S Telzak EE. and INSIGHT SMART Study Group. Factors associated with adherence amongst 5295 people receiving antiretroviral therapy as part of an international trial. J Infect Dis. (2013) 208:40–9. 10.1093/infdis/jis731 23204161 PMC3666133

[27] Cheng Y Nickman NA Jamjian C Stevens V Zhang Y Sauer B. Predicting poor adherence to antiretroviral therapy among treatment-naïve veterans infected with human immunodeficiency virus. Medicine. (2018) 97:e9495. 10.1097/MD.0000000000009495 29480838 PMC5943852

[28] Achappa B Madi D Bhaskaran U Ramapuram JT Rao S Mahalingam S. Adherence to antiretroviral therapy among people living with HIV. N Am J Med Sci 5. (2013) 220–3. 10.4103/1947-2714.109196 23626959 PMC3632027

[29] Shigdel R Klouman E Bhandari A Ahmed LA. Factors associated with adherence to antiretroviral therapy in HIV-infected patients in Kathmandu District, Nepal. HIV/AIDS. (2014) 6:109–116. 10.2147/HIV.S55816 25028564 PMC4077521

[30] Banagi Yathiraj A Unnikrishnan B Ramapuram JT Kumar N Mithra P Kulkarni V. Factors influencing adherence to antiretroviral therapy among people living with HIV in coastal south India. J Int Assoc Provid AIDS Care. (2016) 15:529–33. 10.1177/2325957416661424 27493025

[31] Joseph A Ogah OE Robinson O Matthew NI Ikeola A. Determinants of adherence to antiretroviral therapy among HIV-positive women accessing prevention of mother to child transmission services in Ebonyi State, Nigeria. Ann Med Health Sci Res. (2018) 8:248–53.

[32] Huang Y Zhao N. Generalized anxiety disorder, depressive symptoms and sleep quality during COVID-19 outbreak in China: a web-based cross-sectional survey. Psychiatry Res. (2020) 288:112954. 10.1016/j.psychres.2020.112954 32325383 PMC7152913

[33] Islam MS Ferdous MZ Potenza MN. Panic and generalized anxiety during the COVID-19 pandemic among Bangladeshi people: an online pilot survey early in the outbreak. J Affect Disord. (2020) 276:30–7. 10.1016/j.jad.2020.06.049 32697713 PMC7362838

[34] Chatterjee SS Bhattacharyya R Bhattacharyya S Gupta S Das S Banerjee BB. Attitude, practice, behavior, and mental health impact of COVID-19 on doctors. Indian J Psychiatry. (2020) 62:257–65. 10.4103/psychiatry.IndianJPsychiatry_333_20 32773868 PMC7368446

[35] Zhang J Wang X Jia X Li J Hu K Chen G. Risk factors for disease severity, unimprovement, and mortality in COVID-19 patients in Wuhan, China. lin Microbiol Infect. (2020) 26:767–72. 10.1016/j.cmi.2020.04.012 32304745 PMC7159868

[36] Teo W Yap ES Yip C Ong L Lee CT. The psychological impact of COVID-19 on 'hidden' frontline healthcare workers. Int J Soc Psychiatry. (2021) 284–9. 10.1177/0020764020950772 32779498

[37] Dorward J Khubone T Gate K Ngobese H Sookrajh Y Mkhize S. The impact of the COVID-19 lockdown on HIV care in 65 South African primary care clinics: an interrupted time series analysis. Lancet HIV. (2021) 8:e158–65. 10.1016/S2352-3018(20)30359-3 33549166 PMC8011055

[38] Dear N Duff E Esber A Parikh A Iroezindu M Bahemana E. Transient reductions in HIV clinic attendance and food security during the COVID-19 pandemic for people living with HIV in four African countries. Clin Infect Dis. (2021) 73:10. 10.1093/cid/ciab379 33906235 PMC8135576

[39] Byabene AK Fortes-Déguénonvo L Niang K Manga MN Bulabula A Nachega JB. Optimal antiretroviral therapy adherence as evaluated by CASE index score tool is associated with virological suppression in HIV-infected adults in Dakar, Senegal. Trop Med Int Health. (2017) 22:776–82. 10.1111/tmi.12882 28407436

